# Wearing Garters, Etc., Etc.

**Published:** 1860-10

**Authors:** 


### WEARING GARTERS.

Persons who have nothing but a skinny spindle below the knee, can wear their garters any where, but those who have a real leg, swelling, full, and firm, should wear the garter below the knee, for there it can be kept in position without being so tightened as to interfere with the circulation of the blood, upon which depends the calfy rotundity which adorns the lower—humanity. Several evil results follow a tight-drawn garter above the knee. 1. The leg dwindles in proportion as the blood is detained from it. 2. Free and graceful locomotion is interfered with. 3. Varicose veins form one of the most ugly, unsightly, and intractable of diseases—painful, deforming, and of lifetime duration.

